# Validation of the INDDEX24 mobile app *v*. a pen-and-paper 24-hour dietary recall using the weighed food record as a benchmark in Burkina Faso

**DOI:** 10.1017/S0007114521004700

**Published:** 2022-11-14

**Authors:** Beatrice Rogers, Jérome W. Somé, Peter Bakun, Katherine P. Adams, Winnie Bell, David Alexander Carroll, Sarah Wafa, Jennie Coates

**Affiliations:** 1 Tufts University, Gerald J. and Dorothy R. Friedman School of Nutrition Science and Policy, Boston, MA, USA; 2 Institut de Recherche en Sciences de la Santé, Ouagadougou, Burkina Faso; 3 University of California, Davis, Institute for Global Nutrition, Department of Nutrition, Davis, CA, USA

**Keywords:** Dietary recall, Survey methods, Tablet-based data collection, Accuracy, Cost-effectiveness

## Abstract

Effective nutrition policies require timely, accurate individual dietary consumption data; collection of such information has been hampered by cost and complexity of dietary surveys and lag in producing results. The objective of this work was to assess accuracy and cost-effectiveness of a streamlined, tablet-based dietary data collection platform for 24-hour individual dietary recalls (24HR) administered using INDDEX24 platform *v*. a pen-and-paper interview(PAPI) questionnaire, with weighed food record (WFR) as a benchmark. This cross-sectional comparative study included women 18–49 years old from rural Burkina Faso (*n* 116 INDDEX24; *n* 115 PAPI). A WFR was conducted; the following day, a 24HR was administered by different interviewers. Food consumption data were converted into nutrient intakes. Validity of 24HR estimates of nutrient and food group consumption was based on comparison with WFR using equivalence tests (group level) and percentages of participants within ranges of percentage error (individual level). Both modalities performed comparably estimating consumption of macro- and micronutrients, food groups and quantities (modalities’ divergence from WFR not significantly different). Accuracy of both modalities was acceptable (equivalence to WFR significant at *P* < 0·05) at group level for macronutrients, less so for micronutrients and individual-level consumption (percentage within ±20 % for WFR, 17–45 % for macronutrients, 5–17 % for micronutrients). INDDEX24 was more cost-effective than PAPI based on superior accuracy of a composite nutrient intake measure (but not gram amount or item count) due to lower time and personnel costs. INDDEX24 for 24HR dietary surveys linked to dietary reference data shows comparable accuracy to PAPI at lower cost.

To be effective in addressing nutrition and health challenges, food and nutrition policies must be based on an empirical understanding of food consumption patterns: dietary adequacy, the nutritional value of foods, food safety and food sources. In low- and middle-income countries (LMIC), the framing of food security and nutrition problems increasingly encompasses not only undernutrition but also diet quality^(1)^ and overweight and obesity, with their attendant chronic diseases^(2,3)^. Issues of the safety of the food supply, including microbiological and mycotoxin contamination^(4,5)^ and potentially risky additives^(6)^, are emerging concerns of food policy in LMIC. Detailed information on individual food consumption, collected in a survey that is appropriately representative of the relevant population, is critical for making informed policies and developing effective programmatic responses to assure nutritional health^(7,8)^; lack of individual-level dietary data is a barrier to developing effective solutions^(9)^.

A wide variety of qualitative and semi-quantitative methods are currently used to assess the adequacy of individual dietary intakes in LMIC including dietary diversity scores^(10–15)^ and FFQ^(16,17)^, but their use as a measure of diet quality has been questioned^(18)^, and quantitative estimates yielded by these methods are imprecise^(19–22)^. Household food consumption data derived from household consumption and expenditure surveys have been used to estimate individual consumption but do not account for the intrahousehold allocation of food^(23–25)^ and may miss consumption of food away from home^(26)^. Given these limitations, the detailed information derived from individual quantitative dietary recalls is more appropriate for many types of analyses and policy decisions.

Food policies at the national level manifest their effects at the level of the individual; to make informed food policies, governments need quantitative individual data. Such data allow for measuring important policy-relevant variables, including how foods are prepared (reflecting the preservation of their nutritional quality) and their source (important for understanding potential vehicles for fortification or other interventions and potential contamination risk). In most LMIC, individual quantitative dietary data have been collected infrequently if at all, due to issues related to cost, time, and complexity^(7,27)^.

Quantitative methods for assessing diets include single or multiple 24-hour dietary recalls (24HR), food diaries and weighed food record (WFR)^(28,29)^. The interviewer-administered quantitative 24HR is often viewed as most appropriate for use in dietary surveys, providing more detailed information than the qualitative methods described above; recalls of time periods beyond the previous 24 h are not commonly used as they are subject to serious recall error^(30,31)^. Barriers to use of the 24HR include the time required for the interview, need for more preparation in advance of data collection, more complex data analysis, and the need for a comprehensive set of dietary reference data to convert consumption data into detailed nutrient information.

A 24HR may be administered as a paper-based questionnaire (pen-and-paper interview (PAPI)) or on a tablet or other device (computer-assisted personal interview (CAPI)). Paper-based questionnaires require manual data entry, an error-prone process entailing a long lag between data collection and the availability of results. In contrast, CAPI-based methods have the advantage of electronic data capture and a direct link to reference data such as food composition information, eliminating the need for manual data entry and greatly reducing the time for data processing and analysis.

The INDDEX Project created a mobile data collection application (INDDEX24) for administering a 24HR using the multiple-pass method^(30–33)^, linked to the Global Food Matters Database (FMDB) where the dietary reference data are stored. The foods entered into the recall were drawn from a food list developed in advance and coded so as to link directly to the dietary reference data for analysis; the mobile application provides a standardised electronic data collection platform, customisable to meet the specific needs of a given survey, an architecture that links the data directly to the dietary reference data, and an automated analysis tool that streamlines the production of key results.

The present study reports the results of a comparison of a 24HR administered using a paper-based questionnaire (PAPI) with the CAPI approach using INDDEX24 in terms of accuracy (using the WFR as the benchmark) and cost-effectiveness. Comparisons of time required from initial preparation through production of key results and of cost are reported elsewhere^(34)^. Few studies have compared the 24HR CAPI *v*. PAPI approach in a LMIC context. Several studies have assessed relative cost of 24HR using CAPI *v*. PAPI and identified key cost contributors^(32,35–39)^. None of these studies related cost to the accuracy of the data collected.

## Subjects and methods

### Study setting, design and participants

The study took place in 2019 in Oubritenga province in the Plateau Central region of Burkina Faso. The validation study component was conducted in Moackin village in the rural commune of Absouya, and the interview time assessment study component in Ziniare in the urban commune of Ziniare and in Goue in the rural commune of Loumbila. While the 24HR can be administered as part of a representative survey, the goal of the study was not to represent the population but rather to compare the two administration methods; thus, the study site was purposively selected to be roughly comparable in key socio-demographic characteristics (e.g. economic status and education level) with the average levels in Burkina Faso.

The validation study was a cross-sectional comparative study calibrating two dietary recall methods against a benchmark WFR. The study had two arms. For the first arm (WFR-PAPI), respondents participated in the WFR observation during the first day of data collection and were interviewed about the foods consumed on the observation day using the 24HR PAPI the second day. For the second arm (WFR-INDDEX24), the same procedures were carried out except that participants were interviewed using the INDDEX24 the second day of data collection. To implement the interview time assessment study, a similar design was used with two sub-arms (INDDEX24 and PAPI) but with no WFR for this component.

Participants were women aged 18–49 years living in the study areas. One female member within the eligible age range was enrolled from each household. Exclusion criteria for participation included any mental or physical health issues that could affect the participant’s ability to complete the 1-d observation and the interview. Participants needed to agree to participate in both phases of data collection (observation for WFR and 24HR interview).

### Recruitment and allocation of study participants

A household listing was conducted in Moackin village to identify households with eligible participants and was used to build the sampling frame from which households were randomly selected for the validation study. For the time assessment study, thirty households were systematically selected in each of the two other sites (Ziniare and Goue). In each household, one female member within the eligible age range who was available and consented was enrolled.

For the validation study, participants were contacted by a ‘consenting agent’ who was assisted in locating the selected households by a local guide. The consenting agent first obtained verbal agreement from the household head and then the agent talked with one eligible, available and interested woman of the household to obtain a written consent. Study procedures were clearly explained during the consenting process. Participants were informed that they would be participating in 2 d of data collection, the first for the WFR and the second for the 24HR. After that, the agent scheduled the WFR visit for a day within the next 2 d and arranged the time for the interviewer to arrive before the respondent started preparing the first meal of the day. The consenting agent provided the participant with a bowl and plate, instructed her to use them for taking her meals and explained that she should not change her diet. Equal numbers of participants were recruited in each study arm.

### Data collection

Data collection for WFR and 24HR (INDDEX24 and PAPI) took place in September–October 2019 and was carried out on all days of the week. A total of eight 24HR interviewers and sixteen WFR interviewers were trained for 14 and 10 d, respectively, to collect the data. 24HR interviewers were trained on both INDDEX24 and PAPI and were assigned to conduct one or the other modality alternating by day so that all interviewers conducted interviews using both modalities on all days of the week. Data collection was overseen by two supervisor for the 24HR and three for the WFR. Two individuals trained on 24HR but not retained were hired as consenting agents in charge of participants’ recruitment.

#### Weighed food record

Interviewers arrived at the participant’s house in time to observe the preparation and/or the consumption of the first meal of the day and left after consumption of the last main meal of the evening. Interviewers were trained to remain as discreet and unobtrusive as possible when in the participant’s house between meal preparation and/or consumption periods. They accompanied the participant if she left the house and were trained to record any food consumed away from home. Throughout the WFR observation day, interviewers recorded all details about foods and mixed dishes prepared in the home (quantity of each ingredient, cooking method, total quantity of dish prepared, weight of the pot/pan), quantity of the food consumed by weighing the food and beverages before and after the participant had consumed them using a digital scale (MyWeigh KD 7000 with 1-g accuracy and 7-kg capacity), and time and place of preparation and consumption for all food items. For purchased foods, foods prepared outside of the participant’s household or leftovers from the previous day, the interviewers were instructed to record as many details as possible regarding the food or mixed dish, including the main ingredients, cooking method, and any other distinguishable characteristics, along with quantities. At the end of the WFR observation day, the interviewer arranged with the respondent the time for the 24HR visit the next day and later provided this information to the 24HR team for planning the visit of the 24HR interviewers.

### 24-Hour dietary recall

For the 24HR, a different group of interviewers visited the participant in her house the day after the WFR observation and carried out the 24HR. Before administering the 24HR, interviewers completed the registration of the participant, which consisted of recording a same set of information as for the WFR (e.g. date, household ID, location, birthdate, pregnant/lactating) to link 24HR and WFR data.

For the INDDEX4 interview, interviewers recorded first the full list of all foods, beverages and mixed dishes recalled as consumed by the participant during the previous 24 h and then probed for more details using the food and recipe list developed by the study and integrated in the INDDEX24 app (online Supplemental Information); if the food or recipe was not in the list, the interviewer directly input the required details. Following selection of the food or recipe from the food and recipe list in INDDEX24 (or inputting of a missing food item or non-standard recipe (NSR) name), the amount (portion size) consumed by the participant was estimated using the portion size estimation methods (PSEM) developed by the study team and integrated in the INDDEX24 app (online Supplemental Information). PSEM options used for INDDEX24 included direct weight, life-sized photos, proxy weight using play dough, proxy weight using dried sorghum, proxy weight using water, and standard units. In the case of mixed dishes, interviewers were instructed to select the standard recipe that best fit the description of the mixed dish, based on the participant’s report of ingredients used, with flexibility to accept minor divergences from the standard recipe. The acceptable differences included the addition/subtraction of minor ingredients (e.g. herbs, seasoning and spices), the use of a different type of cooking oil, the omission/addition of oil, and the use of condiments such as tomato, onion, bell pepper generally used in small amounts in the study context. If no close match was found for a reported mixed dish, it was considered as a NSR, and its details (ingredient description and quantities, and total quantity of recipe prepared) were collected by the interviewers with the participants or the dish preparer when possible. If the NSR was like an existing standard recipe, the interviewer could copy some/all the ingredients and use the copied version as a base from which to build the NSR. For recipes purchased or prepared by another person not available to provide details and considered as non-standard, participants were asked to report as many details as possible (ingredients, cooking method, place of purchase/acquisition and whatever other information could be provided).

For 24HR PAPI interview, interviewers used a paper data collection form and printed food and recipe booklets that contained all the same items as the INDDEX24 food and recipe list. During the 24HR PAPI interview, interviewers recorded the detailed description of the food or recipe reported by the participant and identified the best match in the food or recipe books, and then recorded the relevant code on the paper form. If the interviewer could not find a food or recipe in the books, they would describe it in detail and code it as ‘9999’ for food and ‘99 999’ for recipe. The same approach was taken for the standard and NSR selection in INDDEX24, and the same rules were applied to the PAPI data collection. To confirm whether mixed dishes described by the participants were among those listed as ‘standard recipes’, interviewers located the closest recipe in their printed recipe booklet and asked the respondent to list all the ingredients used in preparation of her recipe. Interviewers were trained to consider the recipe as non-standard if there were significant differences between the lists of ingredients (as described earlier); in that case, the recipe details were recorded including the amount prepared, the quantities and description of ingredients used. To estimate amounts consumed of foods and mixed dishes reported, PAPI interviewers had the same list of PSEM options (online Supplemental Information) as for INDDEX24 and were trained to identify which would be the best PSEM option for each food. A list of standard units and their codes was developed for use during PAPI interviews. When a standard unit was selected as the PSEM option, the interviewer asked the respondent about the standard unit size/volume (e.g. small or large bottle of Coca-Cola, French baguette – with weight or volume conversions automatically applied) of the food and the portion of the standard unit they consumed (e.g. 1 unit, half a unit, etc.). Then, interviewers selected the corresponding standard unit code within the list and recorded the code and the portion consumed in the PAPI form.

After the 24HR PAPI and INDDEX24, the interviewers administered the socio-demographic module and concluded the visit. For each day of 24HR data collection, interviewers were assigned two to three respondents with whom to conduct interviews using one type of 24HR modality (either INDDEX24 or PAPI). On average, fourteen WFR observations were conducted per day along with six to eight INDDEX24 and six to eight PAPI interviews following the WFR the next day.

### Outcomes

Food group and nutrient intakes were estimated based on foods, beverages, and mixed dishes consumed and recorded during the WFR and both 24HR modalities (INDDEX24 and PAPI) using nutrient composition from the food composition table compiled by the study team (online Supplemental Information). The FAO/WHO GIFT food groups classification was used to group the foods, beverages and mixed dishes recorded in order to estimate intake by food group. For the time assessment study, the mean 24HR interview time and time spent on individual passes were estimated for both INDDEX24 and PAPI.

### Sample size

**Fig. 1.**
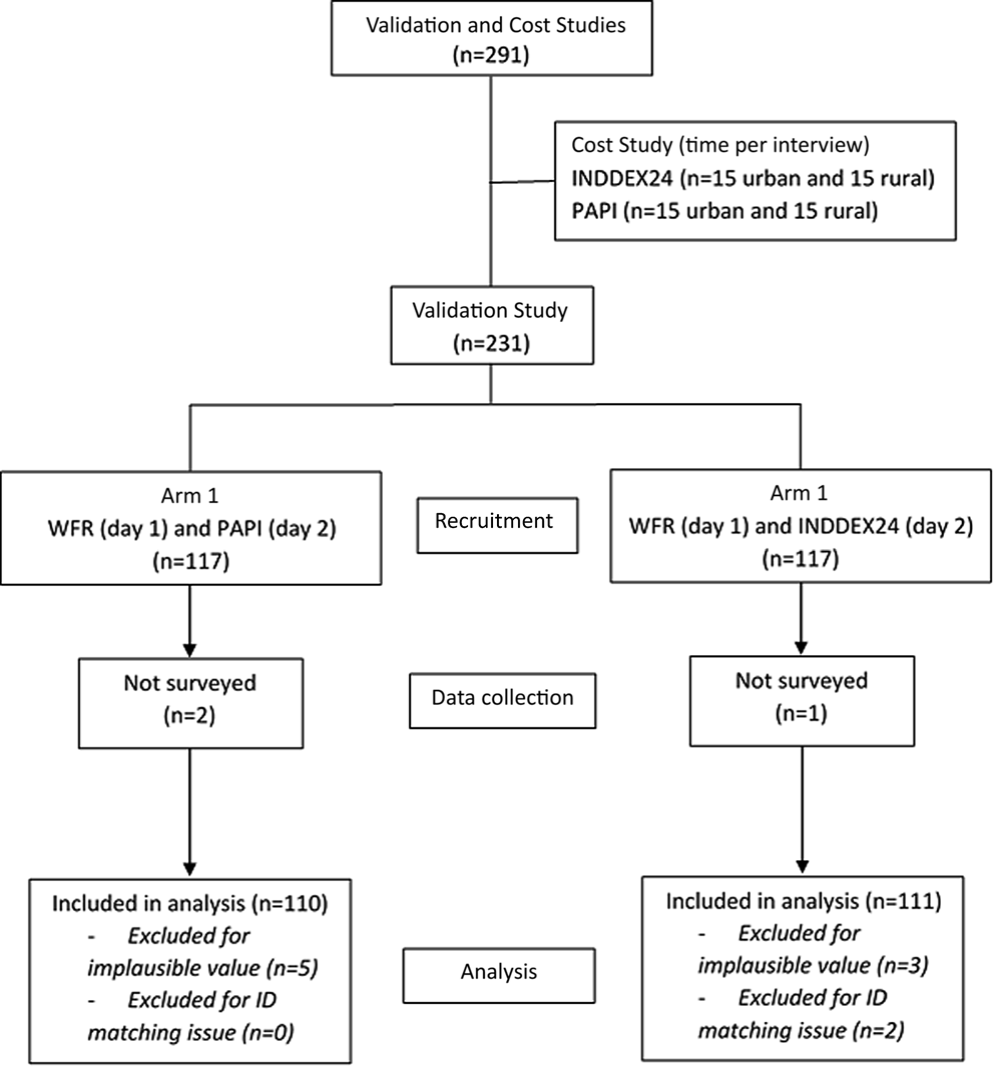
Flow diagram for INDDEX24 validation and cost studies in Burkina Faso. PAPI, pen-and-paper interview; WFR, weighed food record.

The sample size was estimated as follows. First, we assumed that any correlation lower than 0·6 between the WFR-INDDEX24 and WFR-PAPI would be considered unsatisfactory. Second, we considered an increase in correlation of 0·15 or greater (correlation 0·75 or greater) a practically important improvement. Lastly, we set the power at 80 % and *α* level at 5 %. Given these conditions, we estimated the sample size to be 104 for each arm. It was inflated to 117 to account for approximately 12 % non-response. The final number of respondents was 116 for INDDEX24 and 115 for PAPI (Fig. 1).

### Ethical approvals and consent to participate

The study protocol and instruments were approved by the Ethics Committee for Health Research of the Ministry of Health in Burkina Faso and the Institutional Review Board of Tufts University in USA. All participants provided a written informed consent before the enrolment in the study.

### Data management, cleaning and processing

For the INDDEX24, supervisors did some spot checking on tablets immediately after the interview, but most of the review was done by the investigators at the end of daily data collection after exporting the data in CSV. format. INDDEX24 data were reviewed for any outliers in gram weights reported, errors in food categorisation/coding, and completeness of NSR. Supervisors discussed and resolved any issues by early the following day.

Supervisors checked data quality of paper forms for WFR and 24HR PAPI daily, and any issues identified were discussed with the interviewers and resolved. WFR and 24HR PAPI data were entered into a programme built on CSPro version 7.3 using double entry. Hard copies of WFR and PAPI forms were used by the data entry supervisor to resolve any discrepancies. Study coordinators carried out subsequent cleaning to ensure appropriate use of food, recipe and ingredient codes, PSEM codes (PAPI only), value plausibility for amounts/quantities and number of portions, and household ID.

Any new foods or NSR (including food away from home and food prepared on the previous day) or PSEM conversion factors that did not already exist in the INDDEX24 dietary reference data were added to ensure that they had a match with the WFR, PAPI and/or INDDEX24. For the food data processing and conversion into nutrients, the INDDEX24 Global FMDB of dietary reference data (portion conversion factors, standard recipe information and food composition data) was used for WFR and PAPI using SAS v9.4. INDDEX24 analytical reporting feature was used to match all foods and convert them into nutrients automatically. The nutrient intake data sets for WFR, INDDEX24 and PAPI were then merged in order to complete the study analysis.

### Statistical analyses

We conducted a comparison of the socio-demographic characteristics between the two study arms (WFR-INDDEX24 *v*. WFR-PAPI) to check the success of randomisation. Nutrient intake data were checked for outliers by reviewing histograms and scatter plots by nutrient. By convention, energy intakes <2092 kJ or >20920 kJ (< 500 or > 5000 kcal)/d were flagged as outliers^(40–42)^. Any INDDEX4 or PAPI that differed from the respective WFR by > 4184kJ (1000 kcal) in either direction was also flagged for review. Both types of outliers were investigated by looking at the associated WFR or PAPI forms and the INDDEX24 analytical report exports for INDDEX24. Outliers that were due to data entry errors were corrected after reference to the paper forms. Remaining outliers were examined but maintained in the data set. The energy intakes from WFR for three INDDEX24 and five PAPI respondents were flagged as implausible values, and these respondents were excluded from the validation comparison analysis. Shapiro–Wilk tests were used to test for normality, and all nutrients were transformed (log or cube root) to correct for skewness. Nutrients included in the analysis were energy (kJ [kcal]), fat (g), protein (g), carbohydrates (g), total fibre (g), vitamin A (mcg RAE), vitamin C (mg), Ca (mg), Fe (mg) and Zn (mg). Bland–Altman plots were generated to assess visually the agreement between the WFR and the 24HR method for grams consumed, energy, and all nutrients of interest^(43,44)^. Bland–Altman plots show individual differences between the two methods and were used to examine the mean bias, 95 % limits of agreement, and the distribution of bias.

To assess the relative accuracy of the two recall methods, we compared each method to the benchmark (WFR-INDDEX24 and WFR-PAPI) for each nutrient of interest and for the energy contribution from the different FAO/WHO/GIFT food groups. Following the method of Arsenault *et al*.^(45)^, equivalence tests were done using the two one-sided paired *t* test between recall method and WFR at the group level. A 10 % bound was used to assess equivalence, as suggested by other researchers^(45,46)^. A significant *P* < 0·05 for the equivalence test indicates that the WFR is equivalent to the 24HR at 10 % bound; non-significance means the two are statistically significantly different. After equivalence testing, we compared the two recall methods to each other and assessed the relative magnitude of their differences from the WFR using a difference-in-differences approach; comparing the WFR-PAPI difference to the WFR-INDDEX24 difference using a random intercept mixed effects regression model to compare slopes between 24HR methods; this comparison was also done at the group level. In addition, the percentage of INDDEX24 and PAPI respondents with energy and nutrient intakes that fell within specific percentage error categories compared with the WFR was used to assess accuracy of both 24HR modalities at the individual level. This approach allows for assessing the extent to which each modality (INDDEX24 and PAPI) is accurate at the individual level relative to the WFR estimates. All analyses were performed using SAS 9.4 or Stata 15 SE.

### Cost-effectiveness analyses

In order to estimate and compare the cost of using the CAPI modality via INDDEX24 and the PAPI modality to conduct a 24HR and produce a clean, analysable 24 h data set, we conducted a costing study alongside the validation study in Burkina Faso^(34)^. We took an activity- and ingredients-based approach to collecting and processing the cost data for each modality, and costs were collected from a societal perspective, meaning that all costs, including the opportunity cost of respondents’ time, were accounted for^(47)^.

Prior to data collection, we defined the set of activities required to conduct the 24HR and prepare an analysable data set, including the preparation of dietary reference data, survey preparation (including the procurement of supplies and equipment), training, survey execution, data entry, and data cleaning and processing. Then, during the validation study, we recorded the types and quantities of inputs, or ingredients (e.g. personnel, facilities, transportation, equipment and supplies), that were used to carry out each activity. Throughout the validation study, this modality-specific time use and expenditure information was recorded by study personnel using a set of data collection instruments (paper-based quick logs for use in the field, time use logs housed in Google Forms, and Excel-based time and expenditure reporting logs). Time use data for study personnel were valued using wages and salaries, while respondent time was valued at the minimum wage in Burkina Faso^(48)^. All expenditures were converted from West African CFA francs to USA dollars where necessary, adjusted to 2019 USA dollars, and summed in order to estimate the total cost of conducting the 24HR using CAPI and using PAPI. In addition to calculating the total cost of conducting the 24HR using each modality, we also disaggregated costs based on total time (person days), total time costs (person days valued at personnel wage rates/salaries) and total non-time monetary costs.

In addition to the ‘base model’ in which the cost of conducting the 24HR reflected the conditions under which the validation study was conducted, we modelled several cost scenarios to reflect anticipated development of the FMDB. The FMDB, which houses dietary reference data for INDDEX24, is just beginning to be populated with data. However, as future INDDEX24 users share their dietary reference data (e.g. food composition tables, standard recipes, portion conversion factors), other users of the platform may be able to borrow some of these data directly from the FMDB, thereby reducing the costs associated with the preparation of these data for users of INDDEX24. We modelled three alternative scenarios in which we assumed that 25, 50 or 75 % of dietary reference data were borrowed directly from the FMDB for the INDDEX24 modality.

To estimate the cost-effectiveness of using the CAPI modality relative to PAPI, we set these estimates of cost alongside measures of the accuracy of each modality compared with the WFR benchmark. We based our primary measures of cost-effectiveness on three accuracy measures: (1) average percentage accuracy in estimating the number of food items consumed (item count), (2) average percentage accuracy in estimating total gram amount of food intake, and (3) a composite measure of the average percentage accuracy in estimating each of ten nutrients of interest (energy, fat, protein, carbohydrate, fibre, vitamin A, vitamin C, Ca, Fe, and Zn), in the spirit of mean nutrient adequacy described in Hatløy, Torheim, and Oshaug^(13)^ and applied for validation of dietary metrics^(49–51)^.

The average percentage accuracy in estimating the number of food items and gram amount was calculated based on the average percentage error relative to the WFR, that is, we calculated the average percentage error as the absolute value of one minus the ratio of the average estimate based on each 24HR modality to the average estimate based on the WFR, and then average percentage accuracy was calculated as 100 minus the average percentage error. For example, if the average gram amount among the study sample in the PAPI arm was 3500 based in the 24HR and 4000 based on the WFR, then the average PAPI percentage error was calculated as 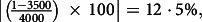 and the average PAPI percentage accuracy, or effectiveness, was 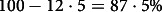 .

For the composite measure of nutrient intake, average percentage accuracy was calculated as the overall average of the average percentage accuracy for each nutrient as described for item count and gram amount. In addition to these primary measures of cost-effectiveness, we used the same methodology to calculate nutrient-specific effectiveness for each of the ten nutrients of interest. The average percentage accuracy of each outcome based on the validation study was assumed to be the same for each of the alternative scenarios.

We calculated cost-effectiveness, or cost per average percentage points of accuracy, based on total cost and each measure of disaggregated cost (time measured in person-days, time cost and non-time (monetary) cost). For the scenarios in which dietary reference data were assumed to be borrowed from the FMDB, effectiveness was based on the average percentage accuracy of each outcome from the validation study.

## Results

The randomisation was well balanced, with no statistically significant differences in participants’ socio-economic and demographic characteristics between the two arms of the validation study (Table 1). A total of 231 women (116 INDDEX24 and 115 PAPI) were enrolled in the validation study in Burkina Faso. Due to implausible values and a matching issue between WFR and INDDEX24 because of errors in respondent ID, five INDDEX24 and five PAPI respondents were excluded from the analysis (Fig. 1). The mean age of participants in the full sample was 29·7 (sd 8·5) years. Most participants (83·5 %) did not have any formal education, and the rest of them had primary or secondary level education. The average household size was 11·7 (sd 5·8). About one-third of participants (35·2 %) were breast-feeding, and 13·4 % were pregnant at the time of the study. Almost all the participants’ households (95 %) owned agricultural land. Borehole was the primary source of drinking water for all the participants. Slightly less than two-thirds of participants (64·7 %) reported preparing food for others all or most of the time. The main household cooking fuel was wood, which was reported by 96·8 % of participants. Participants reported using mainly three types of lighting sources: lamp with rechargeable battery (37·5 %), flashlight with non-rechargeable battery (35·6 %) and solar energy (16·8 %).

**Table 1.** Socioeconomic and demographic characteristics of participants in INDDEX24 validation study in Burkina Faso

	PAPI* n = 115	INDDEX24 n = 116	*P-value*†
Average age (mean (SD)) years	29.8 (8.1)	29.5 (8.8)	*0.776*
Number of people in household (mean (SD))	12.3 (5.8)	11.1 (5.8)	*0.127*
Pregnant respondent (%)	13.9	13.0	*0.361*
Breastfeeding respondent (%)	35.2	39.4	*0.778*
Education level (%)			*0.584*
Primary	11.1	15.5	
Secondary school	2.8	3.6	
High school	–	–	
University	–	–	
Professional	–	–	
No education	86.1	80.9	
Agricultural land possession by household (%)	95.4	94.5	*0.919*
Source of drinking water (%)			*1*
borehole	100	100	
Weekly frequency of food preparation by respondent (%)			*0.999*
All or most of the time	64.8	64.5	
Sometimes	34.2	34.5	
Rarely	1.0	1.0	
Household cooking fuel (%)			*0.228*
Wood	97.2	96.4	
Straw	1.0	1.8	
Gas	0.0	1.8	
Other	1.8	0.0	
Household lightening source (%)			*0.771*
Lamp with rechargeable battery	37.0	34.3	
Flashlight with non-rechargeable battery	37.0	38.0	
Solar energy	15.7	17.6	
Solar-rechargeable flashlight	9.3	8.3	
Other source^‡^	1.0	1.8	
Household floor material (%)			*0.420*
Banco	13.9	9.1	
Cement or concrete	59.3	58.2	
Dirt sand	26.8	32.7	
Household roof material (%)			*0.553*
Metal sheets	81.5	75.5	
Mat or straw	16.7	21.8	
Dirt sand or banco	1.8	2.7	
Household wall material (%)			*0.522*
Earth-made brick or banco	75.0	74.5	
Half banco and half cement	18.5	20.0	
Cement	3.7	4.5	
Other wall material§	2.8	1.0	
Household toilet facility (%)			*0.553*
Pit latrine with slab	22.6	30.0	
Pit latrine without slab	17.9	16.4	
Bush or field	46.2	44.5	
Other toilet facility**	3.9	1.8	
No toilet facility	9.4	7.3	

* PAPI = Pen and Paper Interview

† *P*-value from t-test (binary variables) or chi-2 test (categorical variables) comparison between and PAPI arms

‡ Other lightening sources included generator, woodfire and flashlight from mobile phone

§ Other wall materials included hides/tarpaulin and mat/straw

** Other toilet facilities included ECOSAN latrine, manually poured flush toilet, and simple SanPlat latrine

### Differences in estimates by INDDEX24 *v*. pen-and-paper interview 24-h dietary recall modalities compared with the weighed food record benchmark: nutrient intakes

In general, INDDEX24 and PAPI were equivalent to WFR based on two one-sided paired *t* equivalence test for the item count, gram amount intake and all macronutrient intakes except fat intake for PAPI, as indicated by the significant equivalence test *P*-value (Table 2). Regarding the micronutrient and fibre intakes, neither of the 24HR modalities was equivalent to WFR except for vitamin A and Zn intakes. INDDEX24 and PAPI were equivalent to WFR for vitamin A intake, while only INDDEX24 was equivalent for Zn intake. For the difference-in-differences comparisons, there were very few instances where INDDEX24 and PAPI differed significantly from one another in approximating the WFR estimate except for energy and carbohydrates. For energy intake, INDDEX24 underestimated by 157·8 kcal while PAPI overestimated by 137·8 kcal when compared with WFR. Both INDDEX24 and PAPI underestimated carbohydrate intake, but INDDEX24 underestimated by 73·9 g, while PAPI underestimated by 10 g (Table 2). Thus, for these nutrients (energy, carbohydrates), INDDEX24 and PAPI did not yield comparable results, though both were equivalent to the WFR at the 10 % bound level.

**Table 2.** Comparison of mean differences of INDDEX24 and PAPI to WFR in estimated nutrient consumption for the participants in INDDEX24 validation study in Burkina Faso (Mean values and standard deviations)^*^

	Arm 1: PAPI (*n* 110)							Arm 2: INDDEX24 (*n* 111)							
	WFR^†^		PAPI		WFR-PAPI difference			WFR mean		INDDEX24		WFR-INDDEX24 difference			
	Mean	sd	Mean	sd	Mean	sd	Equivalence test *P*	Mean	sd	Mean	sd	Mean	sd	Equivalence test *P*	Diff-in-diff test (*P*)
Item count	31·1	9·2	23·5	5·4	7·6	7·1	< 0·0001	32·7	10·9	23·9	5·9	8·8	9·2	0·0072	0·4021
Gram amount	4673·5	1324·7	3395·5	1338·6	1278·0	1395·4	< 0·0001	4680·9	1367·3	3360·2	1305·0	1320·7	1495·4	< 0·0001	0·9100
Energy (kcal)	2446·1	906·0	2583·9	1021·8	–137·8	1024·4	< 0·0001	2425·8	956·8	2267·9	1068·1	157·8	1042·5	< 0·0001	0·0305
Fat (g)	47·6	36·0	56·2	39·0	–8·6	39·2	0·0548	45·2	34·7	51·1	38·5	–5·9	37·6	0·0008	0·2688
Protein (g)	78·3	33·2	93·2	41·6	–14·9	44·8	< 0·0001	75·9	34·7	82·8	54·0	–6·9	51·0	< 0·0001	0·0616
Carbohydrates (g)	396·3	144·6	386·3	162·6	10·0	167·0	< 0·0001	396·0	147·6	322·1	145·6	73·9	156·7	< 0·0001	0·0080
Total fibre (g)	39·0	31·4	74·1	43·0	–35·1	43·2	1	40·8	41·2	66·7	61·4	–25·9	63·6	0·9976	0·1068
Vitamin A (mcg RAE)	298·2	320·8	471·8	588·4	–173·6	587·9	0·0123	260·0	208·4	434·6	445·6	–174·7	419·7	0·0083	0·8016
Vitamin C (mg)	62·4	316·2	48·6	45·2	13·8	320·7	0·997	23·3	21·3	46·5	50·8	–23·3	48·4	0·9637	0·3442
Ca (mg)	641·8	587·3	1430·2	1061·1	–788·3	1034·1	0·9342	698·4	665·6	1198·1	1301·9	–499·8	1344·3	0·0545	0·0205
Fe (mg)	21·7	15·4	62·6	59·6	–41·0	58·8	1	22·4	16·6	46·6	37·0	–24·2	36·8	1	0·0199
Zn (mg)	8·6	5·6	15·8	6·9	–7·2	7·4	0·6382	8·4	6·3	13·6	8·8	–5·2	8·8	0·0398	0·3040

* Note the WFR is the minuend, and therefore a negative difference between WFR and 24HR modalities indicates an overestimate, and a positive number indicates an underestimate. Equivalence test *P*-value is from the paired two one-sided t test (TOST) reported using the natural log geometric mean and 10 % bound. A significant *P* < 0·05 for the equivalence test indicates that the WFR is equivalent to the 24HR at 10 % bound; non-significance means the two are statistically significantly different. The difference-in-difference *P*-value is from calculating the difference between each 24HR modality and the WFR and then calculating the overall difference of the differences. A random intercept mixed regression model was used for the difference-in-difference comparison. Statistical significance *P* < 0·05 indicates that the INDDEX24 and PAPI differed from each other with respect to the accuracy of their estimates. FCT completeness for each nutrient based on the foods reported in the WFR: energy (100 %), fat (100 %), protein (100 %), carbohydrates (97·5 %), fibre (92·1 %), vitamin A RAE (98·3 %), vitamin C (84·2 %), Ca (100 %), Fe (99·2 %) and Zn (99·6 %). The distribution of missing FCT values for each nutrient occurred equally across the INDDEX24 and PAPI modalities.

† WFR, weighed food record

For the individual-level comparison, the proportion of INDDEX24 and PAPI respondents with energy intake within 10 % of their WFR estimates was, respectively, 13 and 21 %; 29 and 43 %, respectively, had energy intakes within the 20 % error range (Table 3; see also Bland–Altman plots in online Supplementary Fig. S1–S4). The proportions of respondents with micronutrient intakes within 10 % of their WFR were 20 % or less for all nutrients, with INDDEX24 having slightly lower percentages within ±10 and ±20 percentage points. Both modalities showed a higher percentage of respondents within those error ranges for macronutrients (fat, protein, carbohydrate) than for micronutrients. For the micronutrient intakes, estimates falling within the 10 % error range were under 10 %, and those falling within the 20 % error range were under 20 %, with no consistent difference between modalities. Accuracy of the estimates was even lower for Ca, where less than 5 % of PAPI and INDDEX24 respondents had intakes within 10 % of their WFR estimates; none of the INDDEX24 respondents had vitamin C intake within 10 % of the WFR estimate. Both PAPI and INDDEX24 overestimated intakes far more frequently than they underestimated them at the individual level based on an error range of ± 50 %. This was the case for both macronutrients and micronutrients (online Supplementary Tables S3 and S4).

**Table 3.** Percentage of respondent falling within ranges of percentage error in estimating energy and nutrient intakes with INDDEX24 and PAPI 24HR modalities compared with WFR^*^

	Arm 1: PAPI (*n* 110)						Arm 2: INDDEX24 (*n* 111)					
	0–±10 %	± 10·1–20 %	± 20·1–30 %	± 30·1–40 %	± 40·1–50 %	> ±50 %	0–±10 %	± 10·1–20 %	± 20·1–30 %	± 30·1–40 %	± 40·1–50 %	> ±50 %
Item count	20·9	23·6	20·9	22·7	8·2	3·6	10·8	25·2	26·1	25·2	8·1	4·5
Gram amount	20·0	18·2	12·7	14·5	12·7	21·8	14·4	17·1	15·3	14·4	17·1	21·6
Energy (kcal)	20·9	21·8	10·9	11·8	7·3	27·3	12·6	16·2	14·4	24·3	11·7	20·7
Fat (g)	14·5	15·5	8·2	10·9	11·8	39·1	8·1	9·0	17·1	9·9	6·3	49·5
Protein (g)	17·3	13·6	12·7	8·2	10·9	37·3	10·8	14·4	9·0	12·6	10·8	42·3
Carbohydrates (g)	17·3	13·6	20·0	16·4	12·7	20·0	17·1	11·7	15·3	16·2	17·1	22·5
Total Fibre (g)	5·5	4·5	4·5	6·4	7·3	71·8	9·0	7·2	7·2	8·1	5·4	63·1
Vitamin A (mcg RAE)	4·5	8·2	3·6	9·1	2·7	71·8	7·2	5·4	7·2	7·2	6·9	66·0
Vitamin C (mg)	8·2	7·3	4·5	4·5	6·4	69·1	0·0	4·5	3·6	7·2	7·2	75·7
Ca (mg)	4·5	10·0	3·6	5·5	4·5	71·8	4·5	6·3	8·1	5·4	6·3	69·4
Fe (mg)	2·7	2·7	5·5	2·7	8·2	78·2	7·2	9·9	0·9	3·6	7·2	71·2
Zn (mg)	6·4	8·2	6·4	3·6	6·4	69·1	5·4	12·6	4·5	3·6	9·0	64·0

* FCT completeness for each nutrient based on the foods reported in the WFR: energy (100 %), fat (100 %), protein (100 %), carbohydrates (97·5 %), fibre (92·1 %), vitamin A RAE (98·3 %), vitamin C (84·2 %), Ca (100 %), Fe (99·2 %) and Zn (99·6 %). The distribution of missing FCT values for each nutrient occurred equally across the INDDEX24 and PAPI modalities.

### Differences in estimates by INDDEX24 *v.* pen-and-paper interview 24-h dietary recall modalities compared with the weighed food record benchmark: food group intakes

Table 4 shows the results from the INDDEX24 and PAPI compared with the WFR for the contribution of each food group to total energetic consumption. The largest contribution of energy comes from cereals: about 77 % according to the WFR and 65–66 % for either 24HR modality. The 24HR underestimate was statistically significant for both modalities and comparable between them. The second largest contributor of energy, fats and oils, showed no significant difference between the WFR and either modality, with estimated contributions between 7·1 and 9·2 % of total energy content. The average differences in the contribution of energy from the rest of the food groups were around or less than 5 %. The differences between the WFR and either modality were statistically significant only for vegetables, beverages, spices and condiments, and fish and shellfish. The contribution of beverages and spices/condiments was negligible for WFR and both 24HR modalities; the contribution of vegetables and fish/shellfish was negligible for the WFR but meaningful (around 4 % for fish/shellfish and 6–8 % for vegetables) for 24HR modalities, not different by modality. Overestimates by both 24HR modalities were far more frequent than underestimates (except for cereals), and the two modalities were consistent with each other in overestimating *v*. underestimating the contributions except for the roots and tubers group, which was consumed by only around 5 % of respondents in either study arm. Because small numbers render significance tests unreliable, *P*-values for the differences between the WFR and each 24HR are reported only for those food groups consumed by at least 10 % of respondents.

**Table 4.** Median percentage of energy intake from major FAO/WHO GIFT food groups (Median values and 25th, 75th percentiles)

	Arm 1: PAPI (*n* 110)							Arm 2: INDDEX24 (*n* 111)						
	Respondent consuming (%)	WFR median	WFR 25th, 75th	PAPI median	PAPI 25th, 75th	Median differ-ence	WRS^*^ test *P*	Respondent consuming (%)	WFR median	WFR 25th, 75th	INDDEX24 median	INDDEX24 25th, 75^th^	Median differ-ence	WRS test *P*
Cereals	100	77·4	61·6, 87·1	66·4	55·3, 77·2	11	**0·0001**	100	78·8	61·1, 87·7	65	53·6, 78·1	13·8	**0·0001**
Pulses, seeds and nuts	100	2·8	0·7, 13·8	5·9	3·3, 11·1	–3·1	0·2898	99·1	1·8	0·6, 10·2	4·7	1·3, 8·3	–3·9	0·0549
Vegetables	100	2·6	1·5, 4·8	8·1	3·4, 13·2	–5·5	**0·0001**	100	2·7	1·5, 5·7	6·2	2·4, 13·3	–3·5	**0·0006**
Beverages	100	0·6	0·3, 1·4	0·0	0·0, 0·0	0·6	**0·0001**	100	0·9	0·3, 2·1	0	0, 0	0·9	**0·0001**
Spices and condiments	100	0·2	0·1, 0·4	0·6	0·3, 1·2	–0·4	**0·0001**	100	0·2	0·1, 0·5	0·7	0·3, 1·0	–0·5	**0·0001**
Fish and shellfish	91·8	0·6	0·1, 2·4	4·5	1·4, 7·0	–3·9	**0·0001**	83·8	0·8	0·2, 2·7	4·1	1·9, 8·4	–3·3	**0·0001**
Fats and oils	70·9	8·2	2·5, 14·6	9·2	0·0, 18·9	–1	0·2256	70·3	7·1	2·5, 10·1	8·9	0, 18·6	–1·8	0·9088
Fruits	26·4	2·4	1·3, 4·5	3·4	0·7, 5·2	–1	0·4270	25·2	3·2	1·4, 5·2	4·8	2·3, 7·3	–1·6	0·0872
Sweets and sugars	8·2	1·6	0·5, 2·1	2·1	0·0, 3·9	–0·6	0·8203	16·2	2·3	0, 10·5	3·5	2·4, 6·0	–1·2	0·8145
Roots and tubers	4·5	5·3	2·9, 13·0	2·1	0·0, 11·8	3·2	–	5·4	3·9	1·9, 6·8	7·1	4·6, 8·8	–3·2	–
Meat	3·6	6·0	1·9, 13·5	4·2	1·2, 6·1	1·8	–	6·3	3·6	1·5, 6·2	4·2	1·9, 5·9	–0·6	–
Milk and milk products	0·9	1·7	1·7, 1·7	1·9	1·9, 1·9	–0·2	–	7·2	0	0, 0	3	1·6, 4·5	–3	–
Food for particular nutritional use	0·9	36·5	36·5, 36·5	0·0	0·0, 0·0	0·9	–	0·9	4·4	4·4, 4·4	0	0, 0	4·4	–

* WRS = Wilcoxon rank sum test is a nonparametric test that compares two paired groups. Bolded WRS *P*-value indicates a statistically significant difference between the WFR and the 24HR at or below, *P* = 0·05. Note the WFR is the minuend and therefore a negative difference between WFR and 24HR modalities indicates an overestimate and a positive number indicates an underestimate. Significance of the difference between WFR and 24HR is not shown for food groups consumed by less than 10 % of respondents. These are roots and tubers (5·4 % INDDEX24 and 4·5 % PAPI), meat (6.3% INDDEX24 and 3.6% PAPI), milk and milk products (7·2 % INDDEX24 and 1 % PAPI) and food for particular nutritional use (0·9 % INDDEX24 and 0·9 % PAPI).

### Differences in cost and cost-effectiveness estimates by INDDEX24 *v*. pen-and-paper interview 24-h dietary recall modalities compared with the weighed food record benchmark: food group intakes

The total economic costs of using the INDDEX24 and the PAPI modalities to conduct the 24HR are presented in Table 5. Reflected in the base model, the cost of using INDDEX24 was slightly lower (by $1360) than the cost of using PAPI when no dietary reference data were borrowed from the FMDB. In all four scenarios, while the cost of non-personnel inputs (e.g. supplies, equipment, transport, facilities) was higher for INDDEX24 than PAPI, conducting the 24HR using PAPI was more human-capital intensive, and the higher personnel costs associated with PAPI meant the total cost of INDDEX24 was lower (Table 5).

**Table 5.** Economic cost of conducting a 24HR* using INDDEX24 and PAPI† under alternative scenarios

		Total time	Total time cost	Total non-time cost	Total cost
Scenario	Modality	(person days)	(2019 USD)	(2019 USD)	(2019 USD)
Base model	INDDEX24	463	50,124	27,981	78,105
	PAPI	526	62,162	17,303	79,465
	Difference‡	−63	−12,038	10,678	−1,360
Borrow dietary inputs from FMDB§ (25%)**	INDDEX24	437	47,610	27,175	74,785
	PAPI	526	62,162	17,303	79,465
	Difference‡	−89	−14,552	9,872	−4,680
Borrow dietary inputs from FMDB (50%)**	INDDEX24	412	45,096	26,369	71,466
	PAPI	526	62,162	17,303	79,465
	Difference	−114	−17,065	9,066	−7,999
Borrow dietary inputs from FMDB (75%)**	INDDEX24	386	42,583	25,564	68,146
	PAPI	526	62,162	17,303	79,465
	Difference‡	−140	−19,579	8,260	−11,319

* 24HR, 24-hour dietary recall; FMDB, Food Matters Database.

† PAPI=Pen and Paper Interview

‡ The difference was calculated as INDDEX24-PAPI.

§ FMDB=Food Matters Data Base

** These scenarios assumed that, for the INDDEX24 modality, the specified percentage of dietary reference data (e.g., food composition data, standard recipes, conversion factors) were borrowed from the FMDB.

The average percentage accuracy and cost-effectiveness of each modality based on the three primary measures of effectiveness (item count, gram amount, and a composite measure of nutrient intake) are presented in Table 6. PAPI was slightly more accurate than INDDEX24 (∼1–2 percentage points) for gram amount and item count, while the average percentage accuracy of INDDEX24 based on the composite nutrient intake indicator was ∼9 percentage points more accurate than PAPI. Given the small differences in cost of the two modalities for the base model, PAPI was more cost-effective than INDDEX24 in terms of cost per unit of accuracy in estimating item count, while INDDEX24 was more cost-effective for gram amount and the composite indicator of nutrient intake.

**Table 6.** Cost-effectiveness of conducting a 24HR using INDDEX24 and PAPI in Burkina Faso

				Cost per average percentage accuracy on the basis of:			
			Average percentage accuracy	Total time	Total time cost	Total non-time cost	Total cost
Scenario	Outcome	Modality		(person days)	(2019 USD)	(2019 USD)	(2019 USD)
	Gram amount	INDDEX24	72	6	698	390	1088
Base model		PAPI	73	7	856	238	1094
		Difference*	–1	–1	–157	152	–6
	Item count	INDDEX24	73	6	686	383	1069
		PAPI	76	7	823	229	1052
		Difference	–2	–1	–137	154	17
	Composite nutrient intake†	INDDEX24	48	10	1042	582	1624
		PAPI	39	14	1596	444	2041
		Difference	9	–4	–554	137	–417
Borrow dietary inputs from FMDB (25 %)‡	Gram amount	INDDEX24	72	6	663	379	1042
		PAPI	73	7	856	238	1094
		Difference	–1	–1	–192	140	–52
	Item count	INDDEX24	73	6	651	372	1023
		PAPI	76	7	823	229	1052
		Difference	–2	–1	–171	143	–28
	Composite nutrient intake	INDDEX24	48	9	990	565	1555
		PAPI	39	14	1596	444	2041
		Difference	9	–4	–607	121	–486
Borrow dietary inputs from FMDB (50 %)‡	Gram amount	INDDEX24	72	6	628	367	996
		PAPI	73	7	856	238	1094
		Difference	–1	–2	–227	129	–98
	Item count	INDDEX24	73	6	617	361	978
		PAPI	76	7	823	229	1052
		Difference	–2	–1	–206	132	–74
	Composite nutrient intake	INDDEX24	48	9	938	548	1486
		PAPI	39	14	1596	444	2041
		Difference	9	–5	–659	104	–555
Borrow dietary inputs from FMDB (75 %)‡	Gram amount	INDDEX24	72	5	593	356	949
		PAPI	73	7	856	238	1094
		Difference	–1	–2	–262	118	–144
	Item count	INDDEX24	73	5	583	350	932
		PAPI	76	7	823	229	1052
		Difference	–2	–2	–240	121	–119
	Composite nutrient intake	INDDEX24	48	8	885	531	1417
		PAPI	39	14	1596	444	2041
		Difference	9	–5	–711	87	–624

* The difference was calculated as INDDEX24 minus PAPI.

† Composite nutrient intake is calculated as the overall average of the average percentage accuracy of PAPI (or of INDDEX24) in estimating intake for energy, fat, protein, carbohydrate, fibre, vitamin A, vitamin C, Ca, Fe and Zn relative to intake based on the weighed food record.

‡ These scenarios assume the specified percentage of dietary data inputs (e.g. food composition data, standard recipes, conversion factors) can be borrowed from existing dietary reference data in the INDDEX24 database.

For the modelled scenarios in which 25–75 % of dietary reference data were assumed borrowed from the FMDB for the INDDEX24 modality, the cost of INDDEX24 further decreased relative to PAPI, based on the assumption that the dietary reference data are integrated into the INDDEX24 platform, while the preparation and use of dietary reference data for PAPI would still require implementers to seek out the information and incorporate it into their analysis. Thus, as the percentage of dietary reference data borrowed from the FMBD for INDDEX24 increases, so does the divergence between it and PAPI, since the cost of preparing dietary reference data for PAPI is unchanged. Therefore, INDDEX24 was incrementally more cost-effective than PAPI based on each of the three primary measures of accuracy. Each of these estimates of cost-effectiveness based on time (measured in person days), time cost and non-time monetary cost is available in Table 6.

## Discussion

We hypothesised that the INDDEX24 and PAPI approaches would be comparable in accuracy, and that the time savings resulting from the INDDEX24 approach would mean that cost-effectiveness would favour the CAPI approach, and this hypothesis was largely supported the study.

Beyond the current study and a parallel study in Vietnam, we are unaware of studies that compared the accuracy of a CAPI and PAPI approach using a reference benchmark of accuracy such as the WFR. Some studies have assessed accuracy of one or the other modality using WFR or biomarkers^(52,53)^. Numerous validation studies have reported results similar to the present study – namely, that intakes of many nutrients measured by 24HR were not statistically significantly different from WFR, but that some nutrients did show differences and that some modifications may be needed to reduce error^(54–57)^. But these did not compare one 24HR modality with another, and few were conducted in LMIC. Comparisons of CAPI with PAPI have been done to assess acceptability and feasibility of the methods^(58)^, and differences in measured outcomes^(59)^, but without a benchmark to compare accuracy of the two methods.

A possible limitation of the study is that the comparability of the two modalities may be due in part to the fact that all interviewers conducted both INDDEX24 and PAPI interviews; learning undoubtedly occurred that affected (most likely improved) the quality of the interviews in both study arms. For example, the INDDEX24 platform provided tags and probes as part of the app; interviewers may have learned to ask these questions in the PAPI even when they did not appear in the paper form. Developing the INDDEX24 platform required the systematic collection of dietary reference data prior to survey implementation. These data facilitated the development of an analysable database for both the INDDEX24 and PAPI approaches; such data might not be available in advance in the case of a PAPI as commonly implemented. In particular, the PAPI interviews made use of the repository of standard recipes and followed the same approach to recording NSR using the standard recipes as a basis when possible; this option is not typically available in PAPI dietary surveys.

Another possible limitation is that using the WFR as a benchmark meant that all respondents to the 24HR in the validation study had already been exposed to dietary measurement during the WFR the previous day. This exposure might have increased their awareness of their food consumption, affecting their recall the following day. Previous studies have found that the accuracy of 24HR may be improved by asking respondents to record or otherwise pay attention to their consumption in the days prior to an anticipated dietary survey^(60,61)^, so this ‘priming’ effect may even be an advantage. In the present study, our purpose was not to obtain information about usual or representative diets of the population, but specifically compare the accuracy with which diets were recalled as captured by INDDEX24 and PAPI. While administration of the WFR might have improved the accuracy of the recall, the comparison would not have been possible without administering the WFR as a point of reference, since comparisons were made on detailed consumption information by nutrient and food group.

A final limitation is that the study was implemented in a single geographic area of Burkina Faso, and only to rural women 18–49 years old; the conclusions drawn from the comparison thus may not be applicable to different age or sex groups or to contexts with more varied and complex (e.g. urban) dietary sources. A similar INDDEX24 validation study was conducted in Vietnam, a different geographic context and a country with a more diverse diet, using the same procedures and protocols^(62)^. This second study reached similar conclusions in that both found that INDDEX24 and PAPI performed comparably to each other in terms of accuracy with respect to the WFR. In that study, though, 24HR modalities were equivalent to WFR for more micronutrients, and a larger proportion of respondents fell within ±20 % of the WFR intakes than in the present study.

In addition to accuracy and cost-effectiveness, there are other benefits to using the INDDEX24 platform. It is standardised, so that results from any study using this platform may be comparable with results from other countries or (in the future) other time periods. The INDDEX24 platform is designed to be linked to dietary reference data available in the FMDB. As INDDEX24 becomes more widely used, dietary reference data (e.g. food composition data, portion conversion, standard recipes) will increasingly populate the FMDB, further reducing the initial costs of preparing for a dietary survey and enabling the smooth linkage from survey responses to food composition and other data needed to process the survey results. The intention is to build up the contents of the FMDB and make it widely accessible, allowing faster preparation, survey implementation, analysis and production of usable information. As more dietary reference data can be borrowed from the FMDB, cost-effectiveness becomes even more favourable to INDDEX24. This is important not only to reduce the initial cost of preparation but also to reduce significantly the lag time between completing data collection and producing usable results while they are still relevant. By streamlining the process of conducting individual dietary intake surveys, the intention is that the use of the INDDEX24 platform will contribute to greater availability and use of detailed dietary data to inform effective food and nutrition policies.

### Conclusion

The goal of the study was to compare the INDDEX24 platform, a CAPI approach to conducting individual dietary recall surveys, with the commonly implemented PAPI approach. Results support the conclusion that INDDEX24 is not less accurate than PAPI and that it has distinct advantages in terms of both cost and the timeliness with which survey results can be obtained once data collection is complete. In the future, as the FMDB is populated, the time and cost required for survey preparation using INDDEX24 may be further reduced. Inaccuracies in the estimation of micronutrient consumption and individual-level consumption were comparable between the two modalities; further exploration into ways of increasing the accuracy of recall methods is needed. While innovative technologies for measuring food consumption are under exploration, the 24HR is likely the most feasible method for collecting individual dietary information on populations in LMIC, and the INDDEX24 platform demonstrates advantages that could facilitate such collection and thus encourage the use of dietary surveys in the formation of programmes and policies related to food and nutrition.

## Supplementary material

For supplementary material accompanying this paper visit https://doi.org/10.1017/S0007114521004700. click here to view supplementary material
