# Supplementary material for: Validation of the INDDEX24 mobile app v. a pen-and-paper 24-hour dietary recall using the weighed food record as a benchmark in Burkina Faso
Source: Br J Nutr. 2021 Nov 26;128(9):1817–31. doi: 10.1017/S0007114521004700 (PMC9592947; doi:10.1017/S0007114521004700)
Supplement: Supplementary file 1 [file S0007114521004700sup001.docx]

**Validation of the INDDEX24 mobile app vs. a pen-and-paper 24-hour dietary recall using the weighed food record as a benchmark in Burkina Faso**

Beatrice L Rogers^1*^, Jérôme W Somé^1,2^, Peter Bakun^1^, Katherine P Adams^3^, Winnie Bell^1^, Sarah Wafa^1^, Jennie Coates^1^

# **Supplementary materials**

**Supplemental information on Development of Burkina Faso dietary reference data**

#### Background

The International Dietary Data Expansion Project developed a dietary assessment platform, called INDDEX24 Dietary Assessment Platform (hereafter INDDEX24), in order to increase the availability, access, and use of quantitative dietary data. INDDEX24 is composed of a mobile data collection application for administering a 24HR using the multiple-pass method (Gibson et al., 2017; Gibson & Ferguson, 2008; Harris-Fry et al., 2018; Steinfeldt, Anand, & Murayi, 2013), which is linked to the Global Food Matters Database (FMDB) where the dietary reference data are stored, including the food composition data, conversion factors, edible portions, portion size estimation methods, and standard recipes data, and an automated analysis tool that streamlines the production of key results. In the context of this project, a study was undertaken to assess the accuracy and cost-effectiveness of the INDDEX24 to collect, process and analyze 24-hour dietary recall (24HR) data in comparison with a paper-based approach (PAPI) using weighed food record (WFR) as the benchmark in Burkina Faso. To conduct the study effectively, dietary reference data required for using INDDEX24 to collect, process and analyze 24HR data were developed, including food composition data, information on standard recipes, portion size estimation methods and conversion factors, and food tags and descriptors which are typically used as probes. Appropriate use of probes makes it possible to identify foods and match them to food composition data with a high degree of accuracy. Dietary input development work started in December 2019, several months before INDDEX24 validation study implementation. **Table S1** summarizes the main dietary reference data developed and compiled for the Burkina Faso validation study.

#### Development of food and recipe list

The foods, recipes and ingredients listing (FRIL) was collated from a range of data sources, namely from other researchers who had previously carried out dietary assessment surveys in Burkina Faso, as well as the publicly available food composition tables (FCTs) such as the United Nations Food and Agriculture (FAO)/International Network of Food Data System (INFOODS) Food Composition Table for West Africa 2012 and 2019 (WAFCT 2012 and 2019)^[[1]](#footnote-1)^, with additional foods from the consumption data collected during the INDDEX24 feasibility study (n=60) carried out in 2017. All foods, recipes, and ingredients were compiled in a master FRIL file reviewed by two national food and nutrition experts and the local INDDEX team of investigators, and additional items were added. In addition, they assessed how frequently each food item was likely to be reported in the survey for prioritization purposes. Foods, mixed dishes, and ingredients were grouped based on the classification of FAO/WHO GIFT Food Groups (FAO/WHO) to facilitate the work involved in assessing the listing for completeness and as per the INDDEX24 template requirements.

The local INDDEX team of investigators also determined and assigned the preferred and alternative (when possible or needed) option of portion size estimation methods (PSEMs) to all the foods, recipes, and ingredients. This exercise helped to identify missing density factors and edible portion factors that would be required in order to convert portion sizes into their equivalents in grams.

#### Development of FCT

The list of foods and ingredients from the finalized master FRIL file was used to develop the FCT for INDDEX24 validation study by compiling data from publicly available FCTs such as the United Nations Food and Agriculture (FAO)/International Network of Food Data System (INFOODS) Food Composition Table for Western Africa 2012 and 2019 (WAFCT 2012 and 2019)^[[2]](#footnote-2)^ and USDA National Nutrient Database for Standard Reference Legacy Release, April 2018^[[3]](#footnote-3)^. Primarily, data were compiled from WAFCT 2019 and 2012. When data were not available in the WAFCT 2019 and 2012, data were borrowed from the USDA National Nutrient Database. The INFOODS methods and guidelines were used in the development of the FCT (FAO. (2012). *FAO/INFOODS Guidelines for Food Matching, Version 1.2*. Retrieved from Rome, Italy: http://www.fao.org/docrep/017/ap805e/ap805e.pdf).

#### Compilation of standard recipes

For the Burkina Faso INDDEX24 validation study, the list of recipes was derived from the finalized master FRIL file. Based on this master list, the list of recipes was identified, and experts and the local INDDEX investigators team ranked recipes from ‘Medium’ to ‘High’ priority for developing standard recipes based on the season the of the validation study and the study site location. INDDEX24 follows the methods described by Gibson and Ferguson (2008) for calculating the standard recipes for mixed dishes, allowing ingredient-level analysis useful for deriving food-based indicators. This method requires the individual raw quantity of all ingredients and the total cooked quantity of the recipes to calculate an ingredient fraction, which is then used to derive the quantity of each individual ingredient consumed when a respondent reports consumption of a standard recipe. A total of 84 standard recipes were compiled from existing datasets of dietary reference data of researchers (Yves Martin-Prével and his colleagues from the Institut de Recherche et de Développement (IRD), and Mourad Moursi and his colleague from HarvestPlus) who had previously carried out dietary survey in Burkina Faso (Arsenault et al., 2014). Additionally, 4 variants of 4 standard recipes were developed based on their standard recipes through replacement of major ingredients. For instance, the standard recipe for porridge made with white sorghum was used to create a recipe variant for porridge made with red sorghum flour.

#### Portion size estimation methods (PSEMs) assignment

We preassigned two or at most three PSEM options to the FRIL, with one identified as preferred. For the INDDEX24 validation study in Burkina Faso, we used direct weight, life-sized photos, proxy measures (volume/weight) using sorghum, water, and play dough, and standard unit size as PSEM options (**Table S2**). The general rules that were applied to PSEM assignment were to use direct weight for ingredients and foods usually consumed in raw form and available in households (e.g. corn, sorghum or millet flour, dried groundnuts, etc.); photos for foods that were discrete pieces (e.g. vegetables, fruit, meat, fish) and very commonly consumed mixed dishes (e.g. thick cereal-based porridge, green leafy vegetable sauces, boiled rice, etc.); proxy sorghum for foods or mixed dishes that were heaped or piled (e.g., boiled rice, groundnuts, fresh or dried raw leafy vegetables, etc.); proxy water for large cooked mixed dishes; play dough for foods that could be molded (e.g., vegetables, fruits, chunks of meat or fish); standard unit size for processed and packaged foods (bread, bottled beverages, etc.). All 24HR enumerators used the Elec 3 digital kitchen scale for PSEMs that required weighing (i.e., direct weight and proxy sorghum, water, and playdough).

##### Direct weight

Direct weight was preassigned to many foods (n=669) in the Burkina Faso FRIL. Enumerators were instructed to use the direct weight PSEM method as the primary option unless 1) the item was a mixed dish and manipulating it was not culturally appropriate, or 2) the food in the household was different in any way from what was actually consumed (e.g., the groundnuts available were raw vs. boiled). Enumerators would check if the respondent had the food – as consumed – available in the house and was willing to provide it for the quantification step; if yes, the enumerator would weigh the food and record the gram quantity.

##### Photos and photo substitutes

The INDDEX Project team borrowed validated photos of food portion sizes from a research team that had previously carried out a dietary survey in Burkina (Huybregts et al. 2007). These photos were complemented by an additional set of photos developed by the INDDEX team for a few very commonly consumed mixed dishes, fruits and vegetables, and raw and cooked meat and fish. Enumerators using INDDEX24 used the photo book when the photo PSEM option had been pre-assigned to the food. They would show the photos of the actual food portion sizes to the respondent, or in a few cases the photo of another food used as a substitute because it was very similar in type, content, size, and shape to the food of interest (e.g., different types of fish or cereal-based mixed dishes). When a photo substitute was assigned, the difference was accounted for in the conversion factor by including the density of the substitute food. There was a total of 168 unique photos of foods portion sizes, representing 48 different foods.

##### Proxy sorghum, proxy water and play dough

Foods that did not have a photo and lent themselves to being heaped were matched with the PSEM of proxy sorghum. Play dough was used for whole fruits and vegetables, and chunks of meat or fish. Proxy water was used as one of the PSEMs for estimation of total cooked amount of commonly consumed mixed dishes, in addition to proxy sorghum. For the proxy sorghum and proxy water, enumerators were instructed to ask the respondents to gather the household utensils from which they consumed or prepared a given food and to fill it up to the level that they consumed (removing the left-over amount), or to the level in the pot when they finished preparation (if they were describing the amount prepared). They would then weigh the quantity and record the gram amount. For play dough, enumerators would provide the play dough to the respondent so that she could mold it based on the shape and size of the fruit or chunk of meat she consumed; then, the enumerators would weigh the piece of play dough and record the gram amount, which would then be converted to the appropriate weight of the relevant food. There were 795 conversion factors for proxy sorghum, 110 conversion factors for proxy water, and 208 conversion factors for play dough.

##### Standard unit size

Processed and packaged foods/beverages or foods commonly sold in standard units were matched with the “standard unit” PSEM. Enumerators would ask the respondent about the standard unit size/volume (e.g., small, or large bottle of Coca-Cola, French baguette) of the food and the portion of the standard unit they consumed (e.g., 1 unit, half a unit, etc.). Then, enumerators would select the corresponding standard unit size and record the portion consumed. The standard unit was listed as a PSEM for a total of 169 unique foods/beverages in the Burkina Faso INDDEX24 dietary data inputs database.

#### Development/compilation of portion conversion factors

Except for direct weighing of foods, recipes, and ingredients, all portion size estimation methods required specific conversion factors to allow calculation of grams consumed of food, ingredient, or mixed dish. One or more of the following three types of conversion factors were needed for each item: 1) density factors; 2) edible portion factors; 3) cooking yield factors. PSEMs that required density factors are the ones that use proxy weight methods. Edible portion factors are required when the respondent estimates quantities of foods in forms that include inedible portions (e.g., fruit with peel or pit, fish or meat with bones). Cooking yield factors are needed when the respondent recalls a quantity of raw food (e.g., using photos of raw food) but the food was consumed cooked. Every effort was made to develop the portion conversion factors in advance of the INDDEX24 validation study.

#### Development of food tags and descriptors for probing

The INDDEX24 system relies on tags and descriptors to systematize and standardize the probing process during the 24HR. Foods identified by a ‘base term’ (e.g., milk) would have tags for type (from what animal), full fat or a lower percentage, fresh or canned, for example. Tags would also identify cooking method (e.g., boiled, baked, fried, sautéed, etc.), which affects nutrient content. For INDDEX24, probes were applied using tags and descriptors throughout the food list to ensure the maximal level of information possible. In cases where the respondent did not know the specific details of the food, the enumerator would select the base term as the default for the food match. For the PAPI arm enumerators were trained to ask follow-up questions (i.e., probe) to obtain the same information.

**Supplemental Figures**

Figure S1. Bland-Altman Plot for WFR-PAPI and WFR-INDDEX24 for energy intakes (kcal)


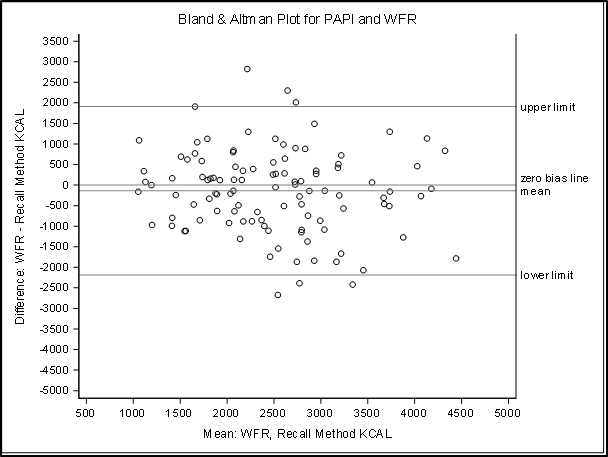

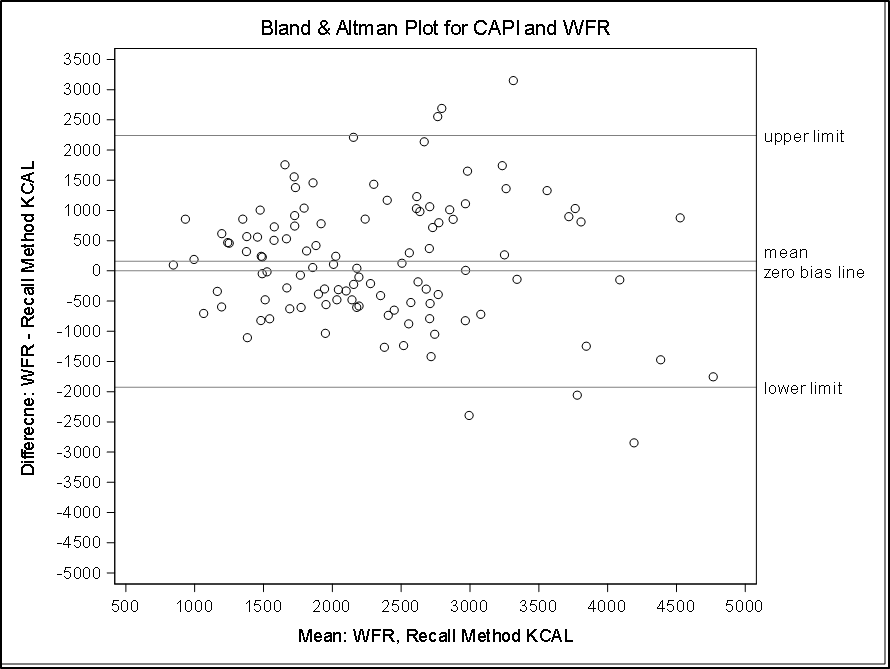


Figure S2. Bland-Altman Plot for WFR-PAPI and WFR-INDDEX24 for fat intakes (gm)


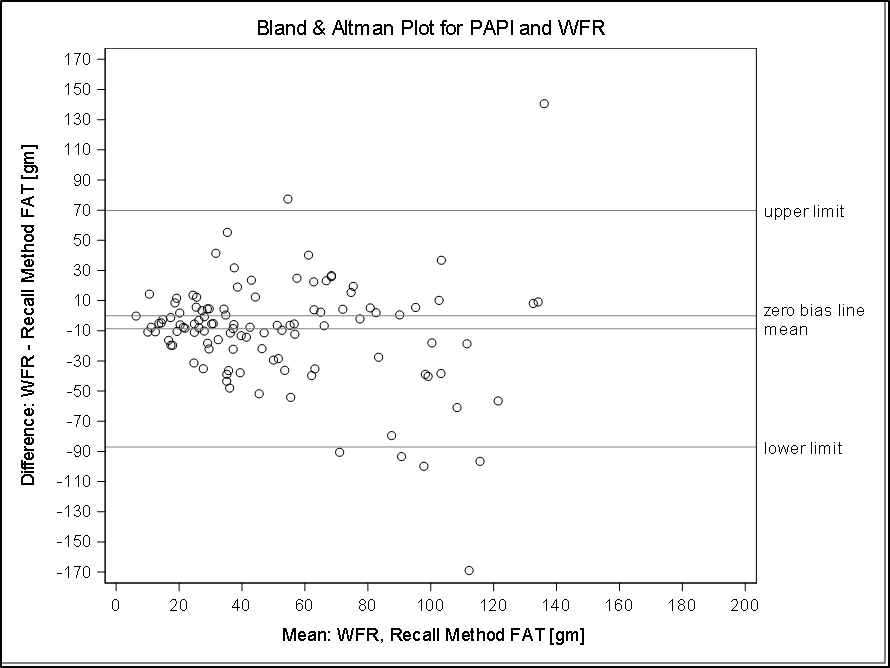

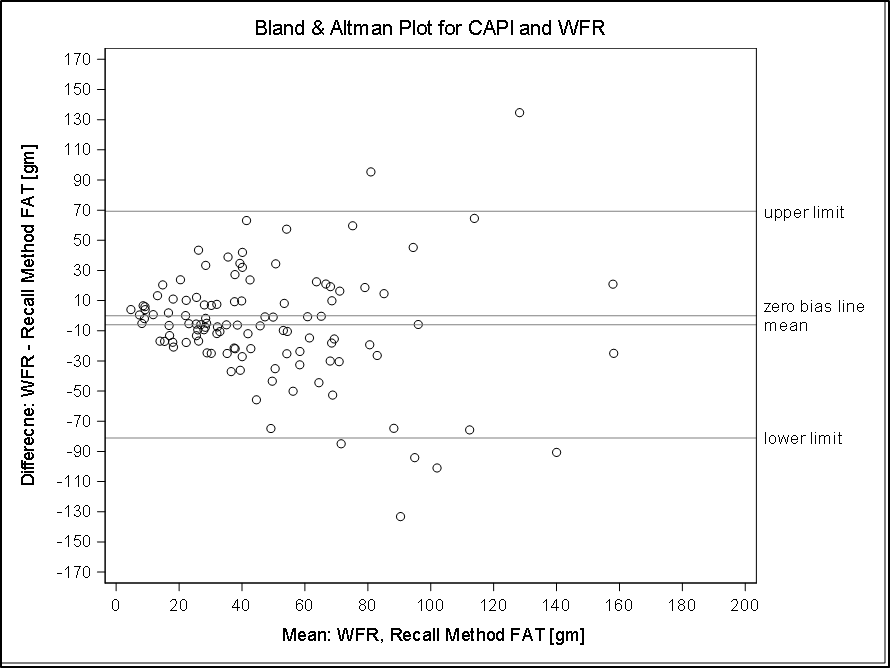


Figure S3. Bland-Altman Plot for WFR-PAPI and WFR-INDDEX24 for protein intakes (gm)


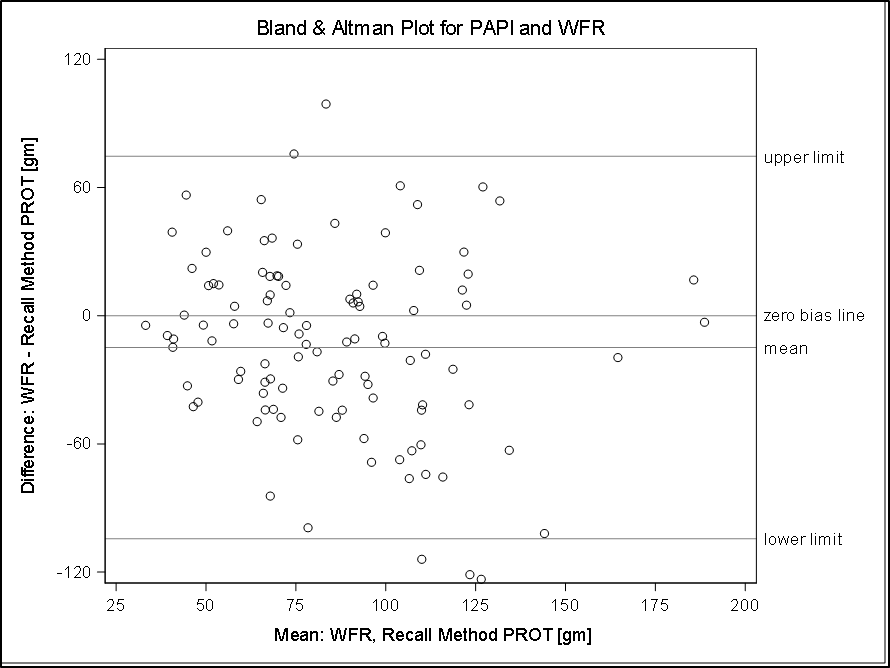

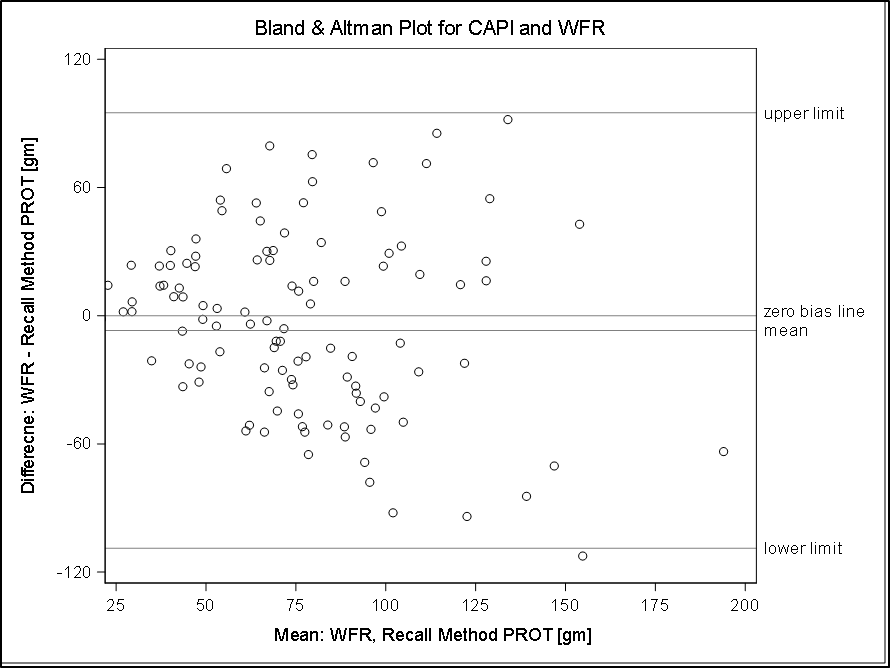


Figure S4. Bland-Altman Plot for WFR-PAPI and WFR-INDDEX24 for carbohydrates intakes (gm)


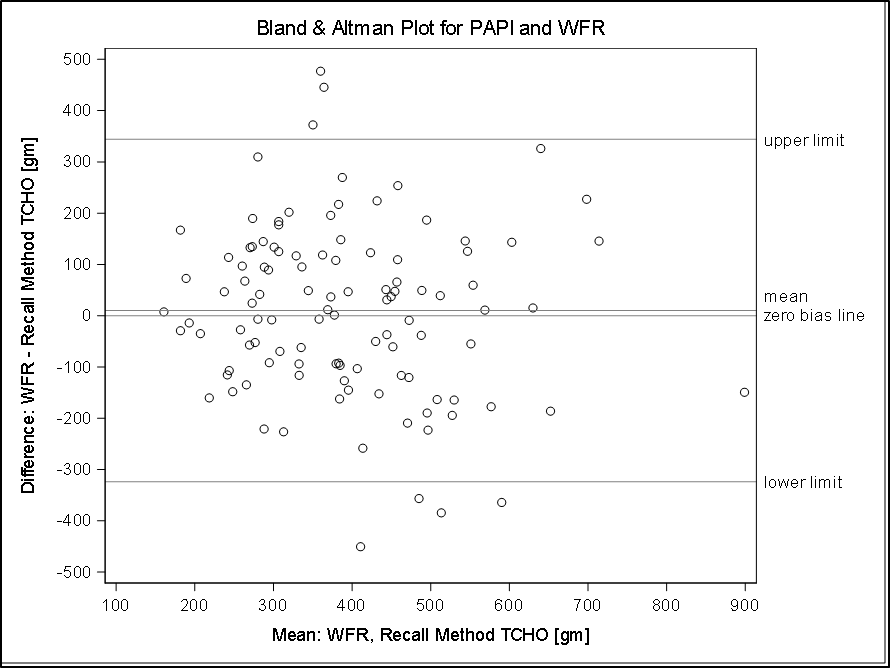

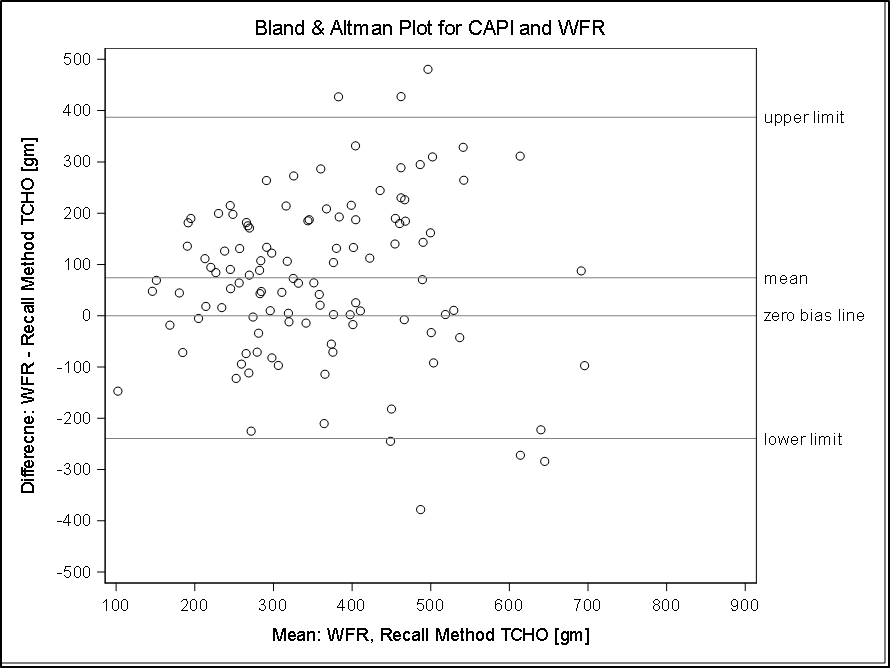


**Supplemental Tables**

Table S1. Dietary reference data developed for INDDEX24 validation study in Burkina Faso

| **Number of foods in Food Composition Table:** | 1,042 foods |
| --- | --- |
| **Primary Food Composition Table source:** | West African Food Composition Table, Food and Agriculture Organization (2012/2019) |
| **Number of food groups:** | 17 foods groups (FAO/WHO GIFT food group classification) |
| **Standard recipes cooked in advance:** | 88 standard recipes |
| **Type and frequency of portion size estimation methods used for foods and standard recipes:** | Direct weight: 669  Photo atlas: 168  Proxy volume (sorghum): 795  Proxy volume (water): 110  Proxy weight (play dough): 208  Standard unit: 169 |
| **Main food probes used in survey** | Cooking method: e.g. raw, boiled, fried, steamed, roasted, grilled  Processing method: e.g. parboiled, fermented, smoked, drained, soaked, bottled, canned  Color: e.g. white, red, purple, brown  Mechanical processing method: e.g. polished, fragmented, refined, crushed, peeled, shelled, cut  Fortification: e.g. fortified, iodized |

Table S2: Type of food and assigned portion size estimation method(s) INDDEX24 validation study in Burkina Faso

| **Type of food** | **Portion size estimation method** |
| --- | --- |
| Ingredients or food items consumed as raw and usually available in household (cereal flour, groundnuts, etc.) | - Direct weight, - Proxy weight with sorghum |
| Leafy vegetables (fresh or dried) | - Direct weight - Proxy weight with sorghum |
| Very commonly consumed mixed dishes | - Proxy weight with sorghum - Photo - Proxy weight with water for mixed dishes prepared in large amount |
| Fruits, vegetables, meat and fish in piece or unit | - Photo - Standard unit - Play dough |
| Processed and packaged foods (bread, bottled beverages, etc.) | - Standard unit |

Table S3. Percent of respondent falling within ranges of percent error in estimating energy and nutrient intakes with PAPI 24HR modality compared to WFR

|  | **< -50%** | **-40 to -50%** | **-30 to -40%** | **-20 to -30%** | **-10 to -20%** | **-10% to 0** | **0 to 10%** | **10 to 20%** | **20 to 30%** | **30 to 40%** | **40 to 50%** | **> 50%** |
| --- | --- | --- | --- | --- | --- | --- | --- | --- | --- | --- | --- | --- |
|  |  |  |  |  |  |  |  |  |  |  |  |  |
| **Item count** | 0 | 0 | 1.82 | 0 | 1.82 | 10 | 10.91 | 21.82 | 20.91 | 20.91 | 8.18 | 3.64 |
| **Gram amount** | 0.91 | 0.91 | 0.91 | 2.73 | 4.55 | 7.27 | 12.73 | 13.64 | 10 | 13.64 | 11.82 | 20.91 |
| **Energy (kcals)** | 21.82 | 3.64 | 5.45 | 3.64 | 10 | 9.09 | 11.82 | 11.82 | 7.27 | 6.36 | 3.64 | 5.45 |
| **Fat (g)** | 32.73 | 8.18 | 3.64 | 4.55 | 10.91 | 3.64 | 10.91 | 4.55 | 3.64 | 7.27 | 3.64 | 6.36 |
| **Protein (g)** | 31.82 | 6.36 | 2.73 | 5.45 | 8.18 | 6.36 | 10.91 | 5.45 | 7.27 | 5.45 | 4.55 | 5.45 |
| **Carbohydrates (g)** | 13.64 | 4.55 | 6.36 | 9.09 | 6.36 | 6.36 | 10.91 | 7.27 | 10.91 | 10 | 8.18 | 6.36 |
| **Total Fiber (g)** | 68.18 | 3.64 | 2.73 | 2.73 | 2.73 | 4.55 | 0.91 | 1.82 | 1.82 | 3.64 | 3.64 | 3.64 |
| **Vitamin A (mcg RAE)** | 42.73 | 0.91 | 0.91 | 2.73 | 4.55 | 4.55 | 0 | 3.64 | 0.91 | 8.18 | 1.82 | 29.09 |
| **Vitamin C (mg)** | 57.27 | 5.45 | 1.82 | 1.82 | 3.64 | 2.73 | 5.45 | 3.64 | 2.73 | 2.73 | 0.91 | 11.82 |
| **Calcium (mg)** | 67.27 | 0 | 0.91 | 1.82 | 4.55 | 1.82 | 2.73 | 5.45 | 1.82 | 4.55 | 4.55 | 4.55 |
| **Iron (mg)** | 76.36 | 6.36 | 0.91 | 1.82 | 0.91 | 0.91 | 1.82 | 1.82 | 3.64 | 1.82 | 1.82 | 1.82 |
| **Zinc (mg)** | 67.27 | 4.55 | 1.82 | 3.64 | 1.82 | 1.82 | 4.55 | 6.36 | 2.73 | 1.82 | 1.82 | 1.82 |
| WFR = weighed food record, PAPI = pen-and-paper interview. FCT completeness for each nutrient based on the foods reported in the WFR: energy (100%), fat (100%), protein (100%), carbohydrates (97.5%), fiber (92.1%), Vitamin A RAE (98.3%) Vitamin C (84.2%), calcium (100%), iron (99.2%), and zinc (99.6%). The distribution of missing FCT values for each nutrient occurred equally across the INDDEX24 and PAPI modalities. | | | | | | | | | | | | |

Table S4. Percent of respondent falling within ranges of percent error in estimating energy and nutrient intakes with INDDEX24 24HR modality compared to WFR

|  | **< -50%** | **-40 to -50%** | **-30 to -40%** | **-20 to -30%** | **-10 to -20%** | **-10% to 0** | **0 to 10%** | **10 to 20%** | **20 to 30%** | **30 to 40%** | **40 to 50%** | **> 50%** |
| --- | --- | --- | --- | --- | --- | --- | --- | --- | --- | --- | --- | --- |
| **Item count** | 0 | 0.9 | 0.9 | 0 | 2.7 | 7.21 | 3.6 | 22.52 | 26.13 | 24.32 | 7.21 | 4.5 |
| **Gram amount** | 1.8 | 0 | 0.9 | 4.5 | 6.31 | 4.5 | 9.91 | 10.81 | 10.81 | 13.51 | 17.12 | 19.82 |
| **Energy (kcals)** | 10.8 | 5.41 | 9.01 | 6.31 | 7.21 | 7.21 | 5.41 | 9.01 | 8.11 | 15.32 | 6.31 | 9.91 |
| **Fat (g)** | 33.33 | 2.7 | 5.41 | 9.01 | 4.5 | 5.41 | 2.7 | 4.5 | 8.11 | 4.5 | 3.6 | 16.22 |
| **Protein (g)** | 28.83 | 4.5 | 3.6 | 4.5 | 4.5 | 5.41 | 5.41 | 9.91 | 4.5 | 9.01 | 6.31 | 13.51 |
| **Carbohydrates (g)** | 9.01 | 2.7 | 3.6 | 2.7 | 3.6 | 7.21 | 9.91 | 8.11 | 12.61 | 12.61 | 14.41 | 13.51 |
| **Total Fiber (g)** | 57.66 | 3.6 | 2.7 | 1.8 | 0.9 | 4.5 | 4.5 | 6.31 | 5.41 | 5.41 | 1.8 | 5.41 |
| **Vitamin A (mcg RAE)** | 44.41 | 1.8 | 2.7 | 0.9 | 1.8 | 3.6 | 3.6 | 3.6 | 6.31 | 4.5 | 5.1 | 21.62 |
| **Vitamin C (mg)** | 60.36 | 1.8 | 1.8 | 0 | 0.9 | 0 | 0 | 3.6 | 3.6 | 5.41 | 7.21 | 15.32 |
| **Calcium (mg)** | 52.25 | 2.7 | 1.8 | 4.5 | 3.6 | 3.6 | 0.9 | 2.7 | 3.6 | 3.6 | 3.6 | 17.12 |
| **Iron (mg)** | 62.16 | 5.41 | 3.6 | 0 | 5.41 | 4.5 | 2.7 | 4.5 | 0.9 | 0 | 1.8 | 9.01 |
| **Zinc (mg)** | 58.56 | 5.41 | 0 | 2.7 | 7.21 | 3.6 | 1.8 | 5.41 | 1.8 | 3.6 | 4.5 | 5.41 |
| WFR = weighed food record, INDDEX24 = INDDEX24 Dietary Assessment Platform. FCT completeness for each nutrient based on the foods reported in the WFR: energy (100%), fat (100%), protein (100%), carbohydrates (97.5%), fiber (92.1%), Vitamin A RAE (98.3%) Vitamin C (84.2%), calcium (100%), iron (99.2%), and zinc (99.6%). The distribution of missing FCT values for each nutrient occurred equally across the INDDEX24 and PAPI modalities. | | | | | | | | | | | | |

1. <http://www.fao.org/infoods/infoods/tables-and-databases/africa/en/> [↑](#footnote-ref-1)
2. <http://www.fao.org/infoods/infoods/tables-and-databases/africa/en/> [↑](#footnote-ref-2)
3. Haytowitz, David B.; Ahuja, Jaspreet K.C.; Wu, Xianli; Somanchi, Meena; Nickle, Melissa; Nguyen, Quyen A.; Roseland, Janet M.; Williams, Juhi R.; Patterson, Kristine Y.; Li, Ying; Pehrsson, Pamela R. (2019). USDA National Nutrient Database for Standard Reference, Legacy Release. Nutrient Data Laboratory, Beltsville Human Nutrition Research Center, ARS, USDA. https://data.nal.usda.gov/dataset/usda-national-nutrient-database-standard-reference-legacy-release. Accessed 2020-08-06. [↑](#footnote-ref-3)
